# Irisin/Integrin αVβ5/AMPK Signaling in Cardiac Fibrosis: Molecular Mechanisms and Therapeutic Potential

**DOI:** 10.3390/cimb48101036

**Published:** 2026-10-06

**Authors:** Darío Cañate Cañate, Patricio Araos, Samir Bolívar González

**Affiliations:** 1 Molecular Pharmacology Laboratory, Research Group in Pharmaceutical Care and Pharmacology, Faculty of Chemistry and Pharmacy, Universidad del Atlántico, Puerto Colombia 081001, Colombia; dacanate@mail.uniatlantico.edu.co; 2 Faculty of Sciences, Universidad San Sebastián, Santiago 7510157, Chile; patricio.araos@uss.cl

**Keywords:** cardiac fibrosis, irisin, AMPK, integrin αVβ5, FNDC5, oxidative stress, TGF-β1, Nrf2, SIRT1

## Abstract

Cardiac fibrosis is a major pathological feature of several cardiovascular diseases and is characterized by excessive extracellular matrix deposition, leading to ventricular stiffening, impaired cardiac function, and ultimately heart failure. Despite its substantial clinical burden, therapies specifically targeting the fibrotic process remain limited. Irisin, a myokine generated by cleavage of fibronectin type III domain-containing protein 5 (FNDC5), has emerged as a cardioprotective factor with potential antifibrotic properties. This review critically examines current evidence on the molecular mechanisms underlying the cardioprotective and antifibrotic effects of irisin, with particular emphasis on integrin αVβ5, AMP-activated protein kinase (AMPK), and their associated signaling pathways. Preclinical studies indicate that irisin engages context-dependent signaling mechanisms that vary according to cell type and pathological setting. These include integrin αVβ5-dependent activation of AMPK or AKT, modulation of oxidative and nitrosative stress, inhibition of ferroptosis through SIRT1/p53 signaling, attenuation of NLRP3 inflammasome activation, preservation of mitochondrial homeostasis, activation of Nrf2-dependent antioxidant responses, and suppression of TGF-β1/Smad2/3-mediated fibroblast activation. Across experimental models, these molecular effects are associated with reduced fibroblast activation and extracellular matrix accumulation, improved mitochondrial and redox homeostasis, and attenuation of pathological cardiac remodeling. Collectively, current evidence identifies irisin as a multifaceted regulator of pathological cardiac remodeling with potential relevance to the attenuation of cardiac fibrosis. However, the available evidence remains predominantly preclinical, and substantial uncertainties regarding receptor and pathway specificity, pharmacokinetics, optimal delivery, safety, and therapeutic efficacy must be resolved before clinical translation.

## 1. Introduction

Cardiovascular diseases remain the leading cause of morbidity and mortality worldwide [1]. Among the pathological processes contributing to disease progression and adverse clinical outcomes, cardiac fibrosis has emerged as a major determinant of ventricular dysfunction and heart failure. This condition is characterized by excessive accumulation of extracellular matrix (ECM) components, predominantly type I and III collagen, within the myocardial interstitium, leading to progressive ventricular stiffening, impaired diastolic function, and, in advanced stages, deterioration of cardiac contractile performance [2,3,4]. Cardiac fibrosis represents a major pathological component of numerous cardiovascular disorders, including hypertensive heart disease, ischemic heart disease, cardiomyopathies, and heart failure [2,3,4,5].

Cardiac fibrosis is a multifactorial process driven by complex interactions among immune cells, cardiomyocytes, endothelial cells, and cardiac fibroblasts, the latter representing the principal effector cells responsible for ECM deposition and scar formation [6,7,8,9]. At the molecular level, mitochondrial dysfunction contributes importantly to disease progression. Excessive production of mitochondrial reactive oxygen species (mtROS) promotes oxidative damage, disrupts cellular bioenergetics, and activates pro-inflammatory and profibrotic signaling pathways, including NF-κB and the NLRP3 inflammasome [10,11,12,13]. In parallel, neurohormonal activation, particularly through the renin–angiotensin–aldosterone system, further amplifies fibrotic remodeling via angiotensin II (Ang II)-mediated stimulation of fibroblast activation, myofibroblast differentiation, and collagen synthesis [2,4,14]. Despite significant advances in understanding the cellular and molecular mechanisms underlying cardiac fibrosis, therapies specifically targeting the fibrotic process remain limited, highlighting the need for novel therapeutic strategies [15,16,17].

Current pharmacological strategies can indirectly attenuate cardiac fibrotic remodeling, particularly through modulation of neurohormonal and hemodynamic pathways. Consequently, increasing attention has focused on molecular pathways directly involved in fibroblast activation, extracellular matrix deposition, and pathological remodeling. Among these, the PTEN/AKT/mTOR axis has emerged as a potential therapeutic target. In an isoprenaline-induced model of cardiac injury, xanthohumol attenuated myocardial hypertrophy and fibrosis, reduced collagen deposition and the expression of α-SMA and collagen I/III, and modulated PTEN/AKT/mTOR signaling [18]. Other experimental approaches target distinct components of the fibrotic response. For example, calcitonin gene-related peptide (CGRP) has been shown to attenuate cardiac fibroblast senescence and the senescence-associated secretory phenotype through upregulation of Klotho [19]. Collectively, these findings illustrate the diversity of molecular pathways that can be targeted to modulate cardiac fibrosis and underscore the rationale for investigating additional endogenous regulators capable of influencing multiple components of pathological cardiac remodeling. 

In recent years, irisin, a myokine generated by proteolytic cleavage of fibronectin type III domain-containing protein 5 (FNDC5), has attracted considerable attention because of its pleiotropic cardioprotective properties [20,21,22,23]. Originally identified as an exercise-induced hormone predominantly secreted by skeletal muscle, irisin is also produced by other tissues and exerts biological effects in multiple organs and cell types [20,21,24]. A major advance in understanding irisin signaling was the identification of αV-class integrins, particularly integrin αVβ5, as functional receptors for irisin [25]. Subsequent studies have implicated αVβ5-dependent signaling in several cardiovascular effects of irisin, although receptor engagement and downstream signaling appear to vary according to cell type and experimental context [24,26].

Accumulating preclinical evidence indicates that irisin exerts cardioprotective and antifibrotic effects through multiple context-dependent signaling mechanisms. Integrin αVβ5 and AMPK have emerged as important components of irisin signaling in specific cardiovascular settings, while additional pathways involving AKT, Nrf2, SIRT1/p53, NLRP3, mitochondrial homeostasis, and TGF-β1/Smad2/3 have been implicated in its biological effects [24,26,27,28,29,30]. Rather than constituting a single conserved signaling cascade, these mechanisms appear to vary according to cell type, pathological stimulus, and experimental model. Collectively, they converge on processes relevant to fibrotic remodeling, including oxidative and nitrosative stress, inflammation, mitochondrial dysfunction, ferroptosis, fibroblast activation, and extracellular matrix accumulation. Direct experimental evidence further supports Nrf2-dependent inhibition of ROS/TGF-β1/Smad2/3 signaling as one mechanism by which irisin attenuates Ang II-induced cardiac fibrosis.

Although previous reviews have addressed the biological and cardiovascular effects of irisin, an integrated analysis of the molecular mechanisms underlying its antifibrotic actions, including the roles of integrin αVβ5, AMPK, and associated signaling pathways, remains limited. Therefore, this review critically examines current preclinical evidence on irisin signaling in cardiac fibrosis, with particular emphasis on integrin αVβ5 and AMPK, their context-dependent downstream mechanisms, and the potential therapeutic implications of targeting irisin-associated pathways for the attenuation of cardiac fibrotic remodeling.

## 2. Pathophysiology of Cardiac Fibrosis

Cardiac fibrosis is a maladaptive remodeling process characterized by excessive accumulation of extracellular matrix (ECM) components, predominantly type I and III collagen, within the myocardial interstitium (Figure 1). This pathological response disrupts normal myocardial architecture, increases ventricular stiffness, impairs electrical conduction, and progressively compromises both diastolic and systolic function, ultimately contributing to the development and progression of heart failure [2,4,31]. Importantly, cardiac fibrosis is not merely a histopathological consequence of myocardial injury but an active determinant of adverse ventricular remodeling. Consistent with the molecular complexity of this relationship, recent bioinformatics analyses of heart failure have identified alterations in cell-death and immune-related gene networks, with enrichment of pathways including TGF-β, Wnt signaling, and renin secretion [32]. Although these transcriptomic associations do not establish a fibrosis-specific mechanism, they further support the convergence of inflammatory, neurohormonal, and profibrotic signaling networks in heart failure progression.

The development of cardiac fibrosis is driven by a complex interplay of inflammatory, metabolic, neurohormonal, and mechanical stimuli. Conditions such as hypertension, myocardial ischemia, diabetes mellitus, obesity, and heart failure promote a persistent state of tissue stress characterized by increased production of reactive oxygen species (ROS), pro-inflammatory cytokines, and growth factors [17,21,24]. These signals activate resident cardiac fibroblasts and stimulate their differentiation into myofibroblasts, the principal effector cells responsible for ECM synthesis and deposition [4,33,34].

Among the molecular pathways involved, transforming growth factor-β1 (TGF-β1) represents a central profibrotic mediator, promoting cardiac fibroblast proliferation, differentiation into myofibroblasts, and extracellular matrix synthesis through canonical Smad-dependent signaling [4,6,23,27]. However, TGF-β1 also engages non-canonical pathways involved in fibroblast activation. In cardiac fibroblasts, TGF-β1 has been shown to downregulate phosphatase and tensin homolog (PTEN), accompanied by increased Akt and mTOR phosphorylation, thereby linking TGF-β1 signaling to the PTEN/Akt/mTOR axis [35]. Pharmacological modulation of this pathway with xanthohumol restored PTEN expression, reduced Akt/mTOR activation, and attenuated TGF-β1-induced fibroblast proliferation, myofibroblast differentiation, and collagen I/III expression [35]. These findings indicate that PTEN/Akt/mTOR signaling represents an additional regulatory mechanism through which TGF-β1 contributes to cardiac fibroblast activation and fibrotic remodeling.

**Figure 1:**
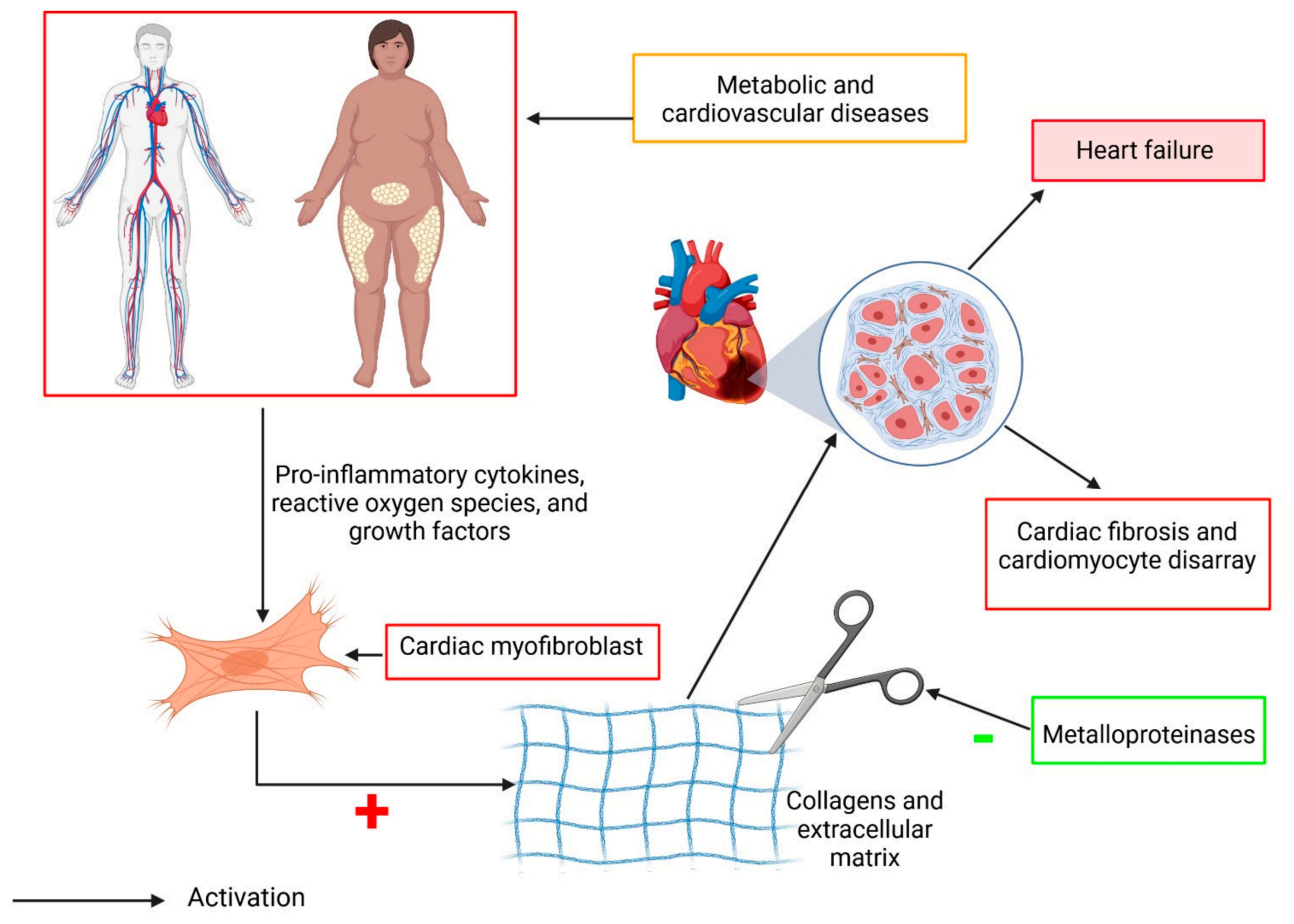
Pathophysiology of cardiac fibrosis. Metabolic and cardiovascular diseases, such as obesity and systemic vascular disorders, generate a state of chronic low-grade inflammation. This state produces and maintains elevated levels of proinflammatory cytokines, reactive oxygen species, and growth factors in the circulation and cardiac tissue [36,37,38,39]. These molecules activate cardiac myofibroblasts, which increase the synthesis and deposition of collagen and other structural proteins in the extracellular matrix [12,13,40]. Matrix metalloproteinases act as negative regulators of this process, degrading some of the deposited collagen. However, when production exceeds degradation, the matrix accumulates excessively, leading to cardiac fibrosis and disorganization of cardiomyocytes [8,15,33]. This progressive fibrosis impairs the heart’s mechanical function, ultimately resulting in heart failure [41,42]. “+” indicates a stimulatory effect; “−” indicates an inhibitory effect. Created in BioRender. Araos, P. (2026) https://BioRender.com/qxnd5kw.

Because these interconnected pathways converge on fibroblast activation, inflammation, oxidative stress, and extracellular matrix accumulation, they constitute attractive therapeutic targets for preventing or attenuating cardiac fibrosis. Consequently, growing attention has focused on endogenous cardioprotective mediators capable of simultaneously modulating multiple profibrotic mechanisms, among which irisin has emerged as a particularly promising candidate.

## 3. Integrin αVβ5 and AMPK in Irisin Signaling

### 3.1. Irisin: Biogenesis and Cardiovascular Functions

Irisin is a cleaved and secreted peptide derived from fibronectin type III domain-containing protein 5 (FNDC5), a transmembrane glycoprotein originally linked to the exercise-responsive PGC-1α signaling program in skeletal muscle [26,43,44,45]. Initially identified as an exercise-induced myokine secreted by skeletal muscle, irisin is now known to be produced by multiple tissues, including adipose tissue, cardiomyocytes, and endothelial cells [46,47,48]. Since its discovery, irisin has attracted considerable attention because of its pleiotropic biological effects, including regulation of energy metabolism, induction of adipose tissue browning, improvement of insulin sensitivity, and modulation of inflammatory responses [21,22,49,50].

Beyond its metabolic functions, accumulating experimental evidence indicates that irisin exerts cardiovascular protective effects. Preclinical studies have demonstrated its ability to attenuate oxidative stress, preserve mitochondrial function, reduce inflammatory signaling, and limit pathological cardiac remodeling in experimental settings including diabetic cardiomyopathy, angiotensin II-induced remodeling, and myocardial infarction [41,51,52]. These findings support a broader role for irisin in the regulation of cardiovascular responses to metabolic, oxidative, and inflammatory stress and provide the basis for investigating its potential contribution to the modulation of pathological cardiac remodeling.

### 3.2. Integrin αVβ5 as a Major Functional Receptor for Irisin

Integrins are heterodimeric transmembrane receptors composed of non-covalently associated α and β subunits that mediate bidirectional communication between cells and the extracellular environment. These receptors play essential roles in cell adhesion, migration, mechanotransduction, proliferation, differentiation, and tissue remodeling. To date, at least 24 distinct integrin heterodimers have been identified in mammals, each displaying specific ligand-binding properties and tissue distribution patterns [53].

Among these receptors, members of the αV integrin family have attracted considerable attention because of their involvement in cardiovascular remodeling and fibrotic diseases. The αV subunit can associate with multiple β subunits, including β1, β3, β5, β6, and β8, generating receptors capable of recognizing extracellular matrix proteins containing the Arg-Gly-Asp (RGD) motif [53]. Among these receptors, integrin αVβ5 has emerged as one of the best-characterized functional receptors mediating the biological effects of irisin.

A major advance in the understanding of irisin signaling occurred when Kim and colleagues identified αV-class integrins as functional irisin receptors and provided biochemical and structural evidence supporting an interaction between irisin and integrin αVβ5 [53,54]. Pharmacological inhibition of αV integrins markedly attenuated irisin-induced signaling and biological responses in osteocytes and adipocytes. Importantly, however, αVβ5 should not be interpreted as the exclusive irisin receptor, because additional αV-containing integrins, including αVβ1, have also shown measurable interaction or functional responsiveness to irisin [54,55]. 

Structurally, integrin αVβ5 consists of αV and β5 subunits, each containing a large extracellular region, a single transmembrane domain, and a short cytoplasmic tail (Figure 2). The extracellular domains mediate ligand recognition and receptor activation, whereas the cytoplasmic regions couple ligand binding to intracellular signaling networks [25,53].

Integrin αVβ5 is expressed in cardiovascular tissues and has been implicated as a functional mediator of irisin signaling in experimental cardiovascular models [24,25,26]. However, evidence demonstrating irisin–αVβ5 signaling is not equally established across cardiovascular cell populations. In experimental diabetic cardiomyopathy, αVβ5-dependent AKT signaling has been implicated in the protective effects of FNDC5/irisin [24], whereas in vascular smooth muscle cells, αVβ5-dependent activation of AMPK and suppression of p38 signaling contribute to protection against Ang II-induced vascular remodeling [26]. Irisin also exerts protective effects in cardiac endothelial cells and fibroblasts through additional signaling mechanisms [27,41]; however, whether αVβ5 represents the predominant functional irisin receptor in these cell populations remains incompletely established. 

Collectively, these observations identify integrin αVβ5 as an important mediator of irisin signaling in cardiovascular tissues. However, the downstream pathways coupled to αVβ5 appear to be cell- and context-dependent. Experimental evidence supports αVβ5-dependent AKT activation in diabetic cardiomyopathy [24] and αVβ5-dependent AMPK activation in Ang II-induced vascular remodeling [26]. Thus, rather than defining a uniform linear signaling cascade, current evidence suggests that irisin engagement of αVβ5 can activate distinct intracellular pathways according to the cellular and pathological context. 

### 3.3. Context-Dependent Activation of AMPK by Irisin

AMP-activated protein kinase (AMPK) is a highly conserved serine/threonine kinase that functions as a central regulator of cellular energy homeostasis and integrates metabolic and stress-related signals to coordinate adaptive responses under conditions of energetic or cellular stress [58]. AMPK is also an important downstream mediator of several biological effects attributed to irisin. Experimental evidence from different tissues indicates that irisin can promote AMPK phosphorylation and activation [22,26,29]; however, the upstream mechanisms responsible for this response appear to vary according to cell type and pathological context rather than constituting a single conserved signaling cascade.

In skeletal muscle cells, experimental evidence indicates that Ca^2+^/CaMKK signaling can contribute to irisin-induced AMPK activation. Irisin increased intracellular Ca^2+^ and AMPK phosphorylation in primary cultured myoblasts, whereas pharmacological inhibition of Ca^2+^/calmodulin-dependent protein kinase kinase (CaMKK) with STO-609 attenuated irisin-induced AMPK activation, supporting the involvement of a Ca^2+^/CaMKK-dependent pathway [59]. In contrast, in human HepG2 cells, irisin-induced AMPK activation was associated predominantly with liver kinase B1 (LKB1) rather than CaMKKβ [60]. Together, these findings indicate that the upstream mechanisms coupling irisin stimulation to AMPK activation are cell-context dependent and should not be interpreted as a single conserved signaling cascade. 

Within the cardiovascular system, experimental evidence linking integrin αVβ5 to AMPK activation has been reported in vascular smooth muscle cells. In Ang II-induced vascular remodeling, irisin activated AMPK and suppressed p38 signaling through an αVβ5-dependent mechanism, whereas blockade of αVβ5 or pharmacological inhibition of AMPK attenuated the protective effects of irisin. The same study showed that irisin improved intracellular Ca^2+^ handling and endoplasmic reticulum homeostasis in vascular smooth muscle cells [26]. Importantly, however, these findings do not establish Ca^2+^ influx through voltage-gated channels or CaMKKβ activation as obligatory upstream steps linking αVβ5 to AMPK in cardiovascular cells.

AMPK activation has also been associated with other cardiovascular effects of irisin/FNDC5. In diabetes-associated atrial remodeling, FNDC5 overexpression was associated with AMPK activation, enhanced antioxidant capacity, improved mitochondrial dynamics, and reduced NLRP3 inflammasome activation [29]. These findings, together with the vascular evidence described above [26], support AMPK as a relevant downstream mediator of irisin/FNDC5 signaling in specific cardiovascular contexts, while indicating that the cellular responses associated with AMPK activation vary according to the experimental model. 

Nevertheless, not all cardiovascular or antifibrotic actions of irisin have been demonstrated to require AMPK. In diabetic cardiomyopathy, FNDC5/irisin-mediated protection has been linked to integrin αVβ5-dependent AKT signaling [24], whereas direct antifibrotic effects in Ang II-induced cardiac fibrosis have been associated with Nrf2-dependent inhibition of ROS/TGF-β1/Smad2/3 signaling [27]. Thus, current evidence supports AMPK as an important, but not universal, signaling node within the broader network of irisin-mediated cardiovascular protection. Rather than a uniform linear αVβ5–AMPK cascade operating across all cardiovascular cell types, irisin appears to engage context-dependent signaling pathways whose relative contribution varies according to cell type, pathological stimulus, and experimental model (Figure 3).

### 3.4. AMPK as an Important Regulator of Antifibrotic Signaling

Beyond its canonical role in cellular energy homeostasis, AMPK has emerged as an important regulator of profibrotic signaling and cardiac fibroblast function. Experimental evidence indicates that AMPK activation can restrain fibroblast proliferation, extracellular matrix production, and cardiac fibrotic remodeling through multiple downstream mechanisms. In experimental cardiac fibrosis, pharmacological activation of AMPK reduced cardiac fibroblast proliferation and collagen production and attenuated interstitial fibrosis [61]. Mechanistically, AMPK activation increased p21 and p27 expression and inhibited CDK2/cyclin E-dependent fibroblast proliferation. In parallel, AMPK activation suppressed HNF-4α-dependent TGF-β1 transcription and increased expression of the antifibrotic miR-29 family [61]. Consistent with these findings, the AMPK activator IMM-H007 attenuated Ang II-induced cardiac fibrosis, reduced fibroblast activation and collagen production, and suppressed TGF-β1/Smad2 signaling, with complementary effects demonstrated in murine and adult human ventricular cardiac fibroblasts [62]. Together, these findings establish AMPK as an important molecular regulator connecting cellular metabolic signaling with mechanisms controlling cardiac fibroblast activation and extracellular matrix accumulation. 

Irisin/FNDC5 can activate AMPK in several experimental settings, including cardiovascular models, as discussed above [26,29]. However, the contribution of AMPK to irisin-mediated cardiovascular protection is context-dependent. In Ang II-induced vascular remodeling, αVβ5-dependent AMPK activation contributes to the protective effects of irisin in vascular smooth muscle cells [26]. In diabetes-associated atrial remodeling, FNDC5 overexpression was associated with AMPK activation, improved mitochondrial dynamics, enhanced antioxidant defenses, and reduced NLRP3 inflammasome activation [29].

Importantly, direct cardiac antifibrotic effects of irisin have also been demonstrated through mechanisms in which AMPK dependence has not been established. In diabetic cardiomyopathy, FNDC5/irisin attenuates myocardial fibrosis and oxidative/nitrosative stress through integrin αVβ5-dependent AKT signaling [24], whereas in Ang II-induced cardiac fibrosis, irisin suppresses fibroblast activation and collagen production through Nrf2-dependent attenuation of ROS/TGF-β1/Smad2/3 signaling [27]. Therefore, AMPK should be considered an important component of the broader signaling network through which irisin exerts cardiovascular protection, rather than an obligatory mediator of all its antifibrotic effects.

## 4. Molecular Mechanisms Underlying the Cardioprotective and Antifibrotic Effects of Irisin

### 4.1. Regulation of Oxidative and Nitrosative Stress 

Oxidative and nitrosative stress are central contributors to the initiation and progression of cardiac fibrosis. Under pathological conditions such as diabetes mellitus, obesity, hypertension, and ischemic heart disease, excessive production of reactive oxygen species (ROS) and reactive nitrogen species (RNS) disrupts cellular homeostasis, promotes inflammatory signaling, and stimulates fibroblast activation and extracellular matrix deposition [13,27,41,63]. Persistent oxidative stress not only damages cardiomyocyte proteins, lipids, and DNA but also amplifies profibrotic pathways, particularly those mediated by transforming growth factor-β (TGF-β), thereby accelerating myocardial remodeling and ventricular dysfunction [23,27].

Accumulating evidence indicates that irisin exerts potent antioxidant and antinitrosative effects through multiple signaling mechanisms. In diabetic cardiomyopathy, one of the principal mechanisms involves integrin αVβ5-dependent AKT signaling and the suppression of inducible nitric oxide synthase (iNOS) and NADPH oxidase 2 (NOX2), two major enzymatic sources contributing to RNS and ROS generation in the stressed myocardium [24]. Elevated iNOS activity promotes excessive nitric oxide production, which reacts with superoxide anions to generate peroxynitrite, a highly reactive oxidant capable of inducing protein nitration, mitochondrial dysfunction, and cellular injury. Simultaneously, activation of NOX2 enhances superoxide generation, further exacerbating oxidative damage and inflammatory responses [24].

Experimental studies have demonstrated that FNDC5/irisin significantly reduces iNOS and NOX2 expression, thereby attenuating ROS and RNS accumulation within cardiomyocytes. This reduction in oxidative burden preserves mitochondrial integrity and limits mitochondria-dependent apoptosis. Importantly, integrin αVβ5-dependent AKT activation appears to mediate these effects in experimental diabetic cardiomyopathy [24].

Beyond direct suppression of oxidant-producing enzymes, irisin can also contribute to the maintenance of mitochondrial function and energy metabolism, thereby reducing mitochondrial ROS generation at its source. Because mitochondria constitute a major intracellular source of ROS in the failing heart, preservation of mitochondrial homeostasis represents an important mechanism through which irisin may limit oxidative injury and interrupt the vicious cycle linking oxidative stress, inflammation, and fibrotic remodeling [41,46,64].

These antioxidant effects are associated with relevant antifibrotic consequences. By reducing oxidative and nitrosative stress, irisin attenuates myocardial fibrosis, cardiomyocyte hypertrophy, and cardiac dysfunction in experimental diabetic cardiomyopathy. Collectively, these findings suggest that suppression of oxidative and nitrosative stress constitutes an important cardioprotective mechanism of irisin, with integrin αVβ5-dependent AKT signaling contributing to the regulation of iNOS/NOX2 in diabetic cardiomyopathy [24] (Figure 4).

### 4.2. Prevention of Ferroptosis Through the SIRT1/p53 Pathway

Ferroptosis is a regulated form of cell death characterized by iron-dependent lipid peroxidation and progressive disruption of membrane integrity. Unlike apoptosis or necrosis, ferroptosis is primarily driven by the accumulation of reactive lipid peroxides resulting from impaired antioxidant defenses. Growing evidence indicates that ferroptosis contributes to the pathogenesis of cardiovascular diseases, including diabetic cardiomyopathy, myocardial infarction, ischemia–reperfusion injury, and cardiac fibrosis [30]. Excessive ferroptotic death of cardiomyocytes can promote the release of damage-associated molecular patterns (DAMPs), amplify local inflammation, and contribute to fibroblast activation and maladaptive cardiac remodeling [4,12,30].

A central regulator of ferroptosis is the glutathione (GSH)-dependent antioxidant system. The cystine/glutamate antiporter system Xc^−^, whose functional light-chain subunit is encoded by SLC7A11, imports extracellular cystine required for intracellular GSH synthesis. Glutathione serves as an essential cofactor for glutathione peroxidase 4 (GPX4), which reduces membrane lipid hydroperoxides and protects cells against ferroptotic death. Impairment of the SLC7A11–GSH–GPX4 axis therefore promotes lipid peroxide accumulation and increases susceptibility to ferroptosis [30].

The tumor suppressor p53 is an important regulator of this pathway. Under specific cellular stress conditions, p53 can repress SLC7A11 transcription, thereby limiting cystine uptake and compromising glutathione-dependent antioxidant defenses. Conversely, SIRT1, an NAD^+^-dependent deacetylase involved in cellular stress responses and metabolic regulation, can modulate p53 activity through deacetylation. This regulatory interaction provides a mechanistic link between SIRT1 activity, p53-dependent control of SLC7A11, and susceptibility to ferroptotic cell death [30].

In experimental diabetic cardiomyopathy, Tang et al. demonstrated that irisin attenuates ferroptosis through modulation of the SIRT1/p53 signaling pathway. Irisin increased SIRT1 expression and reduced p53 acetylation, while restoring SLC7A11 expression, intracellular GSH levels, and GPX4 expression. Importantly, pharmacological inhibition or silencing of SIRT1 attenuated these effects, supporting a functional role for SIRT1 in irisin-mediated regulation of p53 and ferroptosis. Moreover, induction of ferroptosis with erastin diminished the cardioprotective effects of irisin, further supporting ferroptosis inhibition as a relevant component of its protective action in diabetic cardiomyopathy [30].

Collectively, these findings indicate that irisin limits ferroptosis through SIRT1-dependent modulation of p53, thereby preserving the SLC7A11–GSH–GPX4 antioxidant system and reducing lipid peroxidation in cardiomyocytes (Figure 5). By limiting ferroptotic cardiomyocyte injury, this mechanism may contribute to the attenuation of pathological cardiac remodeling observed in experimental diabetic cardiomyopathy [30]. Importantly, although integrin αVβ5 and AMPK participate in irisin signaling in other experimental contexts, their upstream involvement in the SIRT1/p53-mediated anti-ferroptotic mechanism has not been established.

### 4.3. Suppression of Inflammation and NLRP3 Inflammasome Activation

Chronic inflammation is an important contributor to cardiac fibrosis and provides a mechanistic link between cellular injury and maladaptive myocardial remodeling. Following metabolic stress, ischemic injury, or persistent neurohormonal activation, damaged cardiac cells release reactive oxygen species (ROS), mitochondrial DNA, and other damage-associated molecular patterns (DAMPs) that activate innate immune responses within the myocardium [4,10,11,29]. Sustained inflammatory signaling promotes the recruitment and activation of immune cells and the release of cytokines and growth factors that can stimulate fibroblast activation and extracellular matrix deposition [4,9,63,65].

Among the inflammatory pathways implicated in pathological cardiac remodeling, the NLRP3 inflammasome represents an important molecular link between cellular stress, inflammation, and regulated cell death. Activation of NLRP3 promotes assembly of the inflammasome complex with apoptosis-associated speck-like protein containing a CARD (ASC) and procaspase-1, resulting in caspase-1 activation and subsequent maturation of IL-1β and IL-18. Caspase-1 also cleaves gasdermin D (GSDMD), generating its pore-forming N-terminal fragment and promoting pyroptotic cell death. Excessive activation of this pathway can amplify myocardial inflammation and contribute to adverse cardiac remodeling and fibrosis [10,29,37].

Experimental evidence indicates that irisin/FNDC5 can attenuate NLRP3 inflammasome activation in different models of cardiac injury. In diabetes-associated atrial remodeling, reduced FNDC5 expression was accompanied by impaired mitochondrial dynamics, decreased antioxidant capacity, and increased NLRP3 inflammasome activation. Conversely, FNDC5 overexpression increased SOD1 and SOD2 expression, improved mitochondrial membrane potential and mitochondrial dynamics, and reduced NLRP3-mediated inflammation. AMPK signaling was implicated as an important downstream mediator linking FNDC5 to these protective effects [29].

Complementary evidence has been obtained in pressure overload-induced cardiac remodeling. In mice subjected to transverse aortic constriction, irisin treatment reduced myocardial expression of NLRP3, ASC, cleaved caspase-1, and the pyroptosis effector GSDMD-N, together with attenuation of cardiac hypertrophy, myocardial fibrosis, and cardiac dysfunction. Consistent effects were observed in Ang II-treated cardiomyocytes, in which irisin reduced NLRP3 activation, IL-1β, cleaved caspase-1, GSDMD-N, and pyroptotic cell injury [10]. These findings provide direct evidence that suppression of NLRP3-associated pyroptosis contributes to the cardioprotective effects of irisin in pressure overload-induced cardiac remodeling.

Collectively, these studies identify suppression of NLRP3 inflammasome activation as an important component of the cardioprotective actions of irisin/FNDC5. The available evidence suggests that preservation of mitochondrial homeostasis and AMPK-associated signaling may contribute to NLRP3 regulation in diabetes-associated atrial remodeling, whereas direct inhibition of NLRP3-associated pyroptosis has been demonstrated in pressure overload-induced cardiac injury. However, the upstream mechanisms linking irisin to NLRP3 inhibition appear to be context-dependent, and the involvement of integrin αVβ5 has not been established in these experimental models (Figure 6).

### 4.4. Preservation of Mitochondrial Homeostasis and Bioenergetics

Mitochondria are central regulators of cardiomyocyte survival and function owing to their essential role in ATP production, calcium homeostasis, redox balance, and intermediary metabolism. Because the myocardium has one of the highest energetic demands in the body, preservation of mitochondrial integrity is indispensable for maintaining contractile function and preventing pathological cardiac remodeling. Consequently, mitochondrial dysfunction has emerged as a hallmark of numerous cardiovascular disorders, contributing to oxidative stress, metabolic imbalance, inflammation, and ultimately cardiac fibrosis [13,20,45,46].

Under pathological conditions such as diabetes mellitus, obesity, hypertension, and chronic pressure overload, mitochondria undergo profound structural and functional alterations. These include excessive mitochondrial fragmentation, impaired oxidative phosphorylation, reduced ATP synthesis, disruption of mitochondrial membrane potential, and excessive production of mitochondrial reactive oxygen species (mtROS). Together, these abnormalities compromise cellular bioenergetics while simultaneously activating stress-responsive signaling pathways that promote inflammation, cardiomyocyte death, and fibroblast activation [4,12,29].

Accumulating evidence indicates that irisin preserves mitochondrial homeostasis through context-dependent signaling mechanisms, with AMPK emerging as an important mediator of mitochondrial protection in several experimental models. AMPK functions as a master regulator of cellular energy homeostasis by coordinating mitochondrial quality control, metabolic adaptation, and oxidative stress responses. Experimental studies have demonstrated that irisin promotes mitochondrial fusion, limits pathological mitochondrial fragmentation, and preserves mitochondrial network integrity, thereby improving respiratory efficiency and reducing mtROS production [4,27,41,46]. These actions maintain mitochondrial function under metabolic stress and prevent activation of downstream profibrotic signaling pathways.

Beyond its effects on mitochondrial dynamics, irisin also optimizes myocardial bioenergetics. AMPK activation enhances fatty acid oxidation, improves glucose utilization, and supports ATP generation during metabolic stress, allowing cardiomyocytes to maintain energetic homeostasis despite adverse conditions [23,58,59]. Restoration of energy balance reduces susceptibility to oxidative injury and limits the release of inflammatory and profibrotic mediators that drive cardiac remodeling.

An additional mechanism contributing to mitochondrial preservation involves suppression of acyl-CoA:lysocardiolipin acyltransferase 1 (ALCAT1), a mitochondrial enzyme responsible for pathological cardiolipin remodeling. Cardiolipin is a mitochondria-specific phospholipid required for the structural organization and functional stability of the electron transport chain. Under oxidative stress, increased ALCAT1 expression promotes incorporation of highly peroxidizable fatty acids into cardiolipin, destabilizing respiratory chain complexes and amplifying mtROS production. Evidence from extracardiac models further suggests that irisin may regulate mitochondrial phospholipid remodeling. In renal tissue and renal tubular cells exposed to oxidative stress in the setting of myocardial infarction, activation of FNDC5/irisin signaling was associated with AMPK–SIRT1–PGC-1α activation and reduced ALCAT1 expression [45]. Although these findings provide a mechanistic link between irisin signaling, cardiolipin remodeling, and mitochondrial oxidative stress, whether the same ALCAT1-dependent mechanism operates directly in the myocardium remains to be established (Figure 7).

Collectively, these mechanisms position mitochondrial preservation as a central component of the cardioprotective actions of irisin. By regulating mitochondrial dynamics, oxidative metabolism, cellular bioenergetics, and cardiolipin homeostasis through AMPK-associated and other context-dependent mechanisms, irisin contributes to the maintenance of mitochondrial function, reduces oxidative stress, and interrupts multiple upstream events that promote fibroblast activation and extracellular matrix deposition. These mitochondrial actions provide a mechanistic basis through which irisin may contribute to the attenuation of fibrotic cardiac remodeling.

### 4.5. Nrf2 Activation and Inhibition of TGF-β1/Smad Signaling

Transforming growth factor-β1 (TGF-β1) is widely recognized as a master regulator of cardiac fibrosis and represents one of the most important profibrotic cytokines involved in pathological cardiac remodeling [6,27]. In response to myocardial injury, oxidative stress, inflammation, or neurohormonal activation, elevated TGF-β1 signaling promotes the differentiation of resident cardiac fibroblasts into activated myofibroblasts, the primary effector cells responsible for excessive extracellular matrix deposition [23,34]. Persistent activation of this pathway results in progressive accumulation of collagen, ventricular stiffening, and deterioration of cardiac function.

The canonical profibrotic signaling cascade is initiated when TGF-β1 binds to its transmembrane receptors, leading to phosphorylation of Smad2 and Smad3. Activated Smad2/3 subsequently forms a complex with Smad4, which translocates into the nucleus and induces the transcription of genes involved in myofibroblast differentiation, collagen synthesis, fibronectin production, and extracellular matrix remodeling [6,27]. Sustained activation of the TGF-β1/Smad pathway is therefore considered a central molecular event in the development and progression of cardiac fibrosis.

In parallel, oxidative stress plays a critical role in amplifying TGF-β1 signaling. Increased production of reactive oxygen species enhances Smad activation and promotes fibroblast responsiveness to profibrotic stimuli, thereby establishing a positive feedback loop between oxidative stress and fibrogenesis [23,27]. Consequently, molecular pathways capable of simultaneously reducing oxidative stress and suppressing TGF-β1 signaling have emerged as attractive therapeutic targets for preventing cardiac fibrosis.

Among these protective mechanisms, nuclear factor erythroid 2-related factor 2 (Nrf2) has gained considerable attention as a master regulator of cellular antioxidant defenses. Under physiological conditions, Nrf2 remains sequestered in the cytoplasm by Kelch-like ECH-associated protein 1 (Keap1). In response to oxidative stress or specific signaling stimuli, Nrf2 dissociates from Keap1 and translocates to the nucleus, where it promotes transcription of numerous antioxidant and cytoprotective genes, including superoxide dismutase, catalase, glutathione peroxidase, and heme oxygenase-1 [23,27,63]. Through these actions, Nrf2 plays a fundamental role in maintaining redox homeostasis and limiting oxidative tissue injury.

Experimental evidence indicates that irisin activates Nrf2-dependent antioxidant signaling, thereby reducing intracellular ROS accumulation and attenuating ROS-dependent activation of the TGF-β1/Smad2/3 pathway [27]. Importantly, Nrf2 silencing markedly attenuated the antioxidant and antifibrotic effects of irisin, supporting a functional requirement for Nrf2 in this experimental model [27]. Through this mechanism, irisin links enhancement of endogenous antioxidant defenses with suppression of a major profibrotic signaling pathway involved in cardiac fibroblast activation (Figure 8).

The antifibrotic consequences of Nrf2-dependent signaling are reflected in reduced fibroblast activation and extracellular matrix production. By reducing oxidative stress and attenuating TGF-β1/Smad2/3 signaling, irisin limits fibroblast-to-myofibroblast differentiation, decreases α-smooth muscle actin (α-SMA) expression, and reduces collagen production and extracellular matrix accumulation [4,23,27]. These effects contribute to attenuation of Ang II-induced cardiac fibrosis and pathological cardiac remodeling.

Taken together, the available evidence identifies Nrf2-dependent antioxidant signaling and attenuation of TGF-β1/Smad2/3 signaling as important mechanisms underlying the antifibrotic effects of irisin. By enhancing antioxidant defenses and reducing ROS-dependent profibrotic signaling, irisin limits fibroblast activation and extracellular matrix accumulation, thereby contributing to the attenuation of pathological cardiac remodeling [27].

### 4.6. Translation of Mitochondrial Protection into Post-Infarction Cardioprotection

Myocardial infarction remains one of the leading causes of cardiac fibrosis and heart failure worldwide. Although timely restoration of coronary blood flow is essential to salvage ischemic myocardium, reperfusion paradoxically induces additional cellular injury through abrupt oxidative stress, calcium overload, mitochondrial permeability transition, and inflammatory activation. The combined effects of ischemia and reperfusion result in extensive cardiomyocyte death, triggering fibroblast activation, extracellular matrix deposition, scar formation, and progressive ventricular remodeling that ultimately compromises cardiac function [1,4,13,67].

Among the molecular events occurring after myocardial infarction, mitochondrial dysfunction represents an early and important determinant of tissue injury. As discussed in the previous section, impairment of mitochondrial homeostasis leads to ATP depletion, excessive mitochondrial reactive oxygen species (mtROS) production, and activation of multiple forms of regulated cell death. During ischemia–reperfusion, these mitochondrial alterations become markedly amplified, promoting oxidative damage, inflammatory signaling, ferroptosis, apoptosis, and loss of viable cardiomyocytes, thereby creating a highly profibrotic myocardial microenvironment [1,13,45,68].

Within this context, preservation of mitochondrial integrity represents an important mechanism through which irisin may limit myocardial injury and subsequent adverse remodeling. Experimental evidence indicates that irisin can activate AMPK-associated signaling in specific experimental settings, contributing to preservation of mitochondrial function, reduction in mtROS generation, maintenance of cellular bioenergetics, and enhanced cardiomyocyte survival. Consistent with these effects, experimental studies have shown that irisin improves mitochondrial function, suppresses oxidative stress, and attenuates myocardial injury following ischemic stress [28,41,45,51].

Reduced mitochondrial oxidative stress may also attenuate secondary pathological events that follow cardiomyocyte injury. Preservation of mitochondrial function can reduce regulated cell death and the release of damage-associated molecular patterns (DAMPs) and pro-inflammatory mediators, thereby limiting inflammatory responses within the injured myocardium. Because persistent inflammation and cardiomyocyte loss contribute to profibrotic signaling, these effects may indirectly influence fibroblast activation, myofibroblast differentiation, and extracellular matrix deposition during post-infarction remodeling [4,50,52,69].

Accordingly, the cardioprotective effects of irisin may extend beyond acute cytoprotection to influence subsequent myocardial remodeling. Experimental studies have reported reduced myocardial injury and improvements in cardiac function following irisin administration in models of ischemic cardiac injury, while some models also support attenuation of pathological remodeling and fibrosis [10,45,69]. These findings suggest that limiting mitochondrial and cellular injury may contribute to improved structural and functional outcomes following myocardial injury.

Overall, current evidence supports mitochondrial preservation as an important component of irisin-mediated cardioprotection following ischemic myocardial injury. Rather than acting through a single conserved pathway, irisin appears to modulate mitochondrial function, oxidative stress, cellular bioenergetics, regulated cell death, and inflammatory responses through context-dependent signaling mechanisms, including AMPK-associated pathways in specific experimental settings. These convergent effects may contribute to attenuation of adverse ventricular remodeling after myocardial injury, although the receptor and pathway requirements underlying these responses remain incompletely defined.

## 5. Preclinical Evidence Supporting the Antifibrotic Effects of Irisin

Although the molecular mechanisms underlying the cardioprotective actions of irisin have been extensively investigated, the translational relevance of these findings depends largely on the consistency and disease specificity of the available preclinical evidence. Experimental studies conducted in diverse models of cardiovascular injury indicate that irisin modulates several pathological processes associated with cardiac fibrosis, including oxidative stress, inflammation, ferroptosis, mitochondrial dysfunction, fibroblast activation, and extracellular matrix accumulation. However, the strength of the evidence varies across experimental models, and not all reported cardiovascular effects represent direct evidence of myocardial antifibrotic activity.

The preclinical evidence supporting the cardioprotective and antifibrotic actions of irisin derives from complementary in vivo and in vitro experimental systems that reproduce distinct components of cardiac remodeling. Direct evidence of myocardial antifibrotic activity has been obtained in models of diabetic cardiomyopathy, including type 2 diabetic db/db mice and type 1 diabetes induced by streptozotocin, in which irisin attenuated myocardial fibrosis and cardiac dysfunction through mechanisms involving oxidative/nitrosative stress and ferroptosis, respectively [24,30]. Angiotensin II-induced cardiac fibrosis represents another particularly relevant model, as it combines in vivo myocardial remodeling with direct assessment of cardiac fibroblast activation, collagen synthesis, and fibroblast-to-myofibroblast differentiation [27]. Complementary cell-based models, including cardiomyocytes exposed to high glucose and lipid overload and cardiac fibroblasts stimulated with angiotensin II, have enabled more controlled interrogation of the cellular and molecular mechanisms associated with these cardioprotective and antifibrotic effects [24,27,30].

Other studies have examined irisin in diabetes-associated atrial remodeling [29] or Ang II-induced vascular remodeling in vascular smooth muscle cells [26]; although these models provide relevant information on inflammatory, mitochondrial, and vascular mechanisms, they should be distinguished from experimental systems providing direct evidence of myocardial fibrosis. Importantly, several studies have complemented phenotypic observations with pharmacological or molecular pathway manipulation, including receptor/pathway inhibition, gene silencing, and modulation of regulated cell-death mechanisms, thereby providing mechanistic evidence for specific components of irisin signaling (Table 1).

The available preclinical evidence indicates that the effects of irisin involve coordinated activation of protective signaling pathways and suppression of pathological processes associated with cardiac remodeling. Across experimental models, irisin has been associated with activation or restoration of cytoprotective mechanisms, including AKT-, AMPK-, Nrf2-, and SIRT1-dependent signaling, together with attenuation of oxidative and nitrosative stress, inflammatory signaling, ferroptosis, TGF-β1/Smad activation, fibroblast-to-myofibroblast differentiation, and extracellular matrix accumulation [24,27,29,30]. Collectively, these findings suggest that irisin does not act through a single downstream mechanism but rather modulates multiple interconnected pathways involved in myocardial injury and fibrotic remodeling.

Mechanistically, the available studies support integrin αVβ5 and AMPK as important components of irisin signaling, although the downstream pathways engaged appear to depend on the experimental and pathological context. For example, integrin αVβ5-dependent AKT signaling has been implicated in diabetic cardiomyopathy [24], whereas AMPK-associated mechanisms have been described in diabetes-associated atrial remodeling, and αVβ5-dependent AMPK signaling has been demonstrated in Ang II-induced vascular remodeling [26,29]. Other studies demonstrate antifibrotic effects involving Nrf2/TGF-β1/Smad2/3 signaling [27] or SIRT1-dependent regulation of p53 and ferroptosis [30]. Thus, rather than supporting a uniform linear signaling cascade across all models, the preclinical evidence suggests that irisin engages a context-dependent network of cytoprotective and antifibrotic pathways.

Nevertheless, important limitations remain. Most available studies have been conducted in rodent models or isolated cellular systems, which reproduce selected components of human cardiac fibrosis but cannot fully recapitulate its cellular heterogeneity, chronic progression, comorbidities, or complex neurohormonal environment. Moreover, substantial heterogeneity exists across studies regarding disease model, irisin dose, route and duration of administration, and experimental endpoints. Importantly, part of the cardiovascular evidence for irisin derives from models of atrial or vascular remodeling rather than from experimental systems specifically designed to assess myocardial fibrosis [26,29]. Consequently, although current preclinical findings support the antifibrotic potential of irisin, further studies using standardized protocols and clinically relevant chronic cardiac fibrosis models are required before therapeutic translation can be adequately assessed.

### Pharmacological Context of Irisin in Cardiac Fibrosis

The potential therapeutic relevance of irisin should be considered within the broader pharmacological landscape of cardiac remodeling. Established cardiovascular therapies, including renin–angiotensin–aldosterone system (RAAS) inhibitors, mineralocorticoid receptor antagonists (MRAs), angiotensin receptor–neprilysin inhibitors (ARNIs), and sodium–glucose cotransporter 2 (SGLT2) inhibitors, can influence pathways and biological processes associated with fibrotic remodeling through neurohormonal, hemodynamic, metabolic, inflammatory, and extracellular matrix-related mechanisms. Importantly, the evidence supporting effects on myocardial fibrosis differs substantially among these therapeutic classes, ranging from histological assessment of myocardial collagen to circulating markers of extracellular matrix turnover and cardiac magnetic resonance-derived extracellular volume [70,71,72,73]. These therapies have established clinical benefits in their respective cardiovascular indications, but the extent to which direct regression of myocardial fibrosis contributes to these benefits remains incompletely defined and varies according to the therapeutic class and available evidence.

In contrast, experimental strategies such as xanthohumol, calcitonin gene-related peptide (CGRP), and irisin have been investigated for their ability to modulate molecular processes involved in fibrotic remodeling [18,19,35,74,75]. Within this preclinical context, irisin has been reported to modulate multiple interconnected processes, including oxidative and nitrosative stress, inflammation, mitochondrial dysfunction, ferroptosis, and TGF-β1/Smad signaling. However, direct comparative studies between irisin and either established cardiovascular therapies or other experimental antifibrotic strategies are currently lacking. Therefore, the available evidence does not permit conclusions regarding comparative efficacy or therapeutic superiority. Table 2 summarizes the principal mechanistic, evidentiary, and translational differences among these approaches.

## 6. Translational Challenges and Future Perspectives

Despite the compelling preclinical evidence supporting the antifibrotic effects of irisin, several challenges must be addressed before its therapeutic potential can be translated into clinical practice. To date, the vast majority of available data originates from experimental animal models and in vitro studies, whereas clinical evidence in human populations remains extremely limited. Consequently, important questions regarding safety, efficacy, pharmacokinetics, and long-term biological effects remain unresolved. 

One of the principal translational barriers is the incomplete pharmacological characterization of irisin. Experimental pharmacokinetic studies in mice indicate that exogenously administered recombinant irisin has a short circulating half-life, generally below 1 h [76,77]. More recent quantitative analyses reported plasma half-lives of approximately 0.3–0.4 h across recombinant irisin doses of 100–1000 μg/kg, whereas fusion to an albumin-binding domain prolonged systemic exposure to approximately 10 h [77]. These findings indicate that rapid clearance may represent an important limitation for therapeutic development, while also demonstrating that molecular engineering can substantially modify irisin pharmacokinetics. At the same time, the biogenesis of endogenous irisin remains incompletely resolved. Although FNDC5 undergoes signal-peptide processing and cleavage of its extracellular domain, the precise sequence of maturation, cleavage, and release of endogenous human irisin requires further clarification [78,79]. Consequently, pharmacokinetic properties determined using recombinant irisin in experimental animals should not be assumed to reflect the kinetics of endogenous irisin in humans.

Safety and dose optimization represent additional unresolved issues. Preclinical studies have employed heterogeneous irisin doses, administration routes, and treatment schedules, ranging from repeated systemic administration in cardiovascular disease models to engineered long-acting formulations designed to prolong circulating exposure [30,77]. This heterogeneity, together with the limited pharmacokinetic characterization of native irisin, does not yet permit definition of an optimal therapeutic dose, dosing interval, or exposure threshold. Evidence from non-cardiac tissues further indicates that the biological effects of irisin may depend on the dose and administration regimen. In bone, low-dose intermittent irisin administration has been associated with increased cortical bone mass, whereas studies using different exposure paradigms have demonstrated effects on osteocyte signaling and sclerostin expression, underscoring the need to characterize dose- and regimen-dependent responses more systematically [76,80]. Importantly, systematic toxicological studies defining the therapeutic window, maximum tolerated dose, and adverse-effect profile of chronic pharmacological irisin administration remain lacking. Moreover, systemic administration is not expected to produce cardiac-restricted exposure. Pharmacokinetic studies demonstrate distribution of exogenous irisin to multiple extracardiac tissues, including the lung, liver, and kidney, while αV-containing integrins mediate irisin signaling in other responsive tissues, including bone and adipose tissue [76,78]. The biological and safety consequences of prolonged systemic irisin exposure therefore remain insufficiently characterized. These considerations underscore the need for systematic dose–response, pharmacokinetic, biodistribution, immunogenicity, and repeated-dose toxicology studies before chronic irisin-based therapy can be considered for clinical evaluation.

Another important challenge concerns the biological complexity of cardiac fibrosis. Fibrotic remodeling arises from interacting mechanisms involving oxidative stress, inflammation, mitochondrial dysfunction, neurohormonal activation, fibroblast phenotypic transitions, and extracellular matrix remodeling. Although irisin appears capable of modulating several of these processes experimentally, whether these effects can be reproduced in the heterogeneous clinical settings associated with human cardiac fibrosis remains uncertain. This complexity underscores the need to determine which molecular components of irisin signaling are conserved across disease etiologies and which are dependent on the specific pathological context.

Cross-organ comparisons may provide additional insights into conserved and tissue-specific mechanisms of fibrotic remodeling. Recent evidence from pulmonary fibrosis identified a PARP1–FOXN3–p38 regulatory axis in which PARP1 stabilizes FOXN3, thereby restraining Smad signaling and myofibroblast activation; disruption of this feedback mechanism promotes a profibrotic response [81]. Although these findings suggest potential convergence with TGF-β/Smad-dependent mechanisms involved in cardiac fibrosis, the role of the PARP1–FOXN3–p38 axis has not been established in cardiac fibroblasts or myocardial fibrosis. Therefore, whether this regulatory network operates in the heart or interacts with irisin-dependent signaling remains to be determined experimentally. Comparative studies across fibrotic organs may help identify conserved molecular nodes while distinguishing tissue-specific mechanisms relevant to future antifibrotic strategies.

An additional translational consideration concerns the receptor specificity of irisin. Although integrin αVβ5 is currently the best-characterized irisin-binding integrin, available evidence does not support its interpretation as an exclusive receptor. Biochemical and functional studies have shown that irisin can interact with αV-containing integrins, with αVβ5 representing the most extensively characterized interaction, while αVβ1 has also shown measurable interaction and functional responsiveness [76]. Importantly, much of the receptor-level evidence has been obtained in non-cardiovascular experimental systems, particularly osteocytes and adipocytes, indicating that the relative contribution of individual integrin heterodimers may depend on cell type, integrin expression profile, and tissue context [76]. Therefore, whether αVβ5 represents the predominant functional irisin receptor in cardiac fibroblasts, cardiomyocytes, and other myocardial cell populations remains incompletely established. This lack of absolute receptor specificity may also have translational implications, because systemic administration of irisin or irisin-based therapeutics could engage αV-containing integrins in extracardiac tissues. Future studies should therefore define the binding affinity, receptor occupancy, and downstream signaling associated with distinct integrin heterodimers in cardiac versus extracardiac cells before receptor-targeted therapeutic strategies are pursued.

Despite these uncertainties, the biological actions of irisin provide several potential avenues for therapeutic development. In addition to recombinant irisin, future strategies could explore FNDC5-based approaches, engineered irisin analogues, or peptide-based formulations designed to improve stability, bioavailability, or tissue exposure. Pharmacological modulation of receptor-associated or downstream signaling pathways may represent an additional strategy; however, such approaches will require a clearer understanding of receptor specificity, tissue-dependent signaling, and potential off-target effects before therapeutic selectivity can be established.

Advances in molecular engineering and drug-delivery technologies may provide opportunities to overcome limitations associated with the short circulating half-life and systemic delivery of exogenous irisin. An engineered albumin-binding domain–irisin fusion protein substantially prolonged the plasma half-life of irisin and increased systemic exposure in mice [77]. In a separate preclinical study, irisin conjugated to PEGylated gold nanoparticles produced greater cardioprotective effects than soluble irisin in mice with sepsis-associated cardiac dysfunction, including improved cardiac function and reduced apoptosis and inflammatory responses [82]. However, neither approach has demonstrated selective delivery to fibrotic myocardium, and strategies that prolong systemic exposure may also increase irisin exposure in extracardiac tissues. Development of cardiac-targeted delivery systems could therefore represent a potential strategy to improve tissue selectivity, but this approach requires direct experimental validation in models of cardiac fibrosis. Combination strategies with established cardiovascular therapies that exert fibrosis-modulating effects, including RAAS inhibitors, mineralocorticoid receptor antagonists, ARNIs, and SGLT2 inhibitors, also warrant investigation. Future studies should determine whether irisin provides additive or complementary antifibrotic effects when combined with these therapies rather than assuming therapeutic synergy.

In advanced or end-stage heart failure, heart transplantation may become necessary in selected patients, shifting therapeutic priorities from modulation of native myocardial remodeling toward immune regulation and prevention of allograft rejection. Recent experimental evidence indicates that T-cell-specific DNMT1 deletion reduces CDKN1A promoter methylation, promotes CD4+ T-cell senescence, and prolongs cardiac allograft survival [83]. Although mechanistically distinct from irisin-mediated antifibrotic signaling, these findings illustrate how therapeutic targets change across the progression from potentially modifiable cardiac remodeling to end-stage disease requiring organ replacement.

Finally, the integration of bioinformatics, multi-omics approaches, and artificial intelligence/machine learning (AI/ML) may provide additional opportunities for the clinical translation of irisin-based strategies. Transcriptomic and other omics datasets can be interrogated to identify molecular signatures associated with fibrotic remodeling and heart failure, whereas machine-learning approaches may facilitate biomarker selection and validation and the stratification of patients according to molecular or imaging phenotypes [84]. Such approaches are already being explored for biomarker discovery in heart failure and for the detection of myocardial fibrosis using electrocardiographic and imaging-derived features [85]. In the context of irisin, integration of circulating irisin levels with molecular markers related to its downstream signaling networks, extracellular matrix turnover, inflammatory profiles, and quantitative imaging parameters could help define patient subgroups in which irisin-associated pathways are most relevant. However, these applications remain prospective, and no validated AI/ML model currently identifies irisin-responsive cardiac fibrosis phenotypes or predicts therapeutic response to irisin. Future studies should therefore integrate well-characterized clinical cohorts with multi-omics and imaging data and incorporate rigorous external validation before such computational approaches can inform patient selection or therapeutic development.

Collectively, current preclinical evidence supports irisin as a multifunctional regulator of molecular processes involved in cardiac remodeling and fibrosis. However, successful clinical translation will require rigorous characterization of its pharmacokinetics, dose–response relationships, safety, receptor specificity, and tissue-dependent signaling, followed by validation in clinically relevant disease models and ultimately in human studies. Targeted delivery strategies, biomarker-guided patient selection, and computational approaches may complement this translational pathway, but their utility in irisin-based therapy remains to be established experimentally and clinically.

## 7. Conclusions

Cardiac fibrosis remains a major contributor to adverse cardiac remodeling, ventricular dysfunction, and heart failure, yet therapies specifically targeting the fibrotic process remain limited. The evidence reviewed in this article identifies irisin as a multifunctional cardioprotective factor capable of modulating several molecular pathways involved in fibrotic progression. Rather than acting through a single linear signaling cascade, current evidence indicates that irisin engages context-dependent mechanisms involving integrin αVβ5, AMPK, AKT, SIRT1/p53, Nrf2, NLRP3, and TGF-β1/Smad signaling. Through these context-dependent mechanisms, irisin has been associated with reduced oxidative and nitrosative stress, ferroptosis, inflammatory signaling, fibroblast activation, and extracellular matrix accumulation, together with preservation of mitochondrial homeostasis and attenuation of pathological cardiovascular remodeling.

Evidence from multiple preclinical models therefore supports a role for irisin as a multifaceted regulator of pathological cardiac remodeling. Importantly, however, the relative contribution of individual receptors and downstream signaling pathways appears to vary according to cell type, pathological stimulus, and experimental model. This mechanistic heterogeneity should be considered when interpreting the antifibrotic effects attributed to irisin and when defining potential molecular targets for therapeutic intervention. Nevertheless, the ability of irisin to modulate several processes involved in fibrotic remodeling provides a rationale for further investigation of its therapeutic potential in cardiac fibrosis.

Despite these promising findings, substantial translational challenges remain. Current evidence is predominantly preclinical, while the pharmacokinetics, optimal dose and route of administration, tissue-specific receptor engagement, long-term safety, and therapeutic efficacy of exogenous irisin remain insufficiently characterized. Future studies should therefore prioritize rigorous mechanistic validation in clinically relevant models, pharmacokinetic and safety characterization, development of targeted delivery strategies, and ultimately evaluation in human studies. Collectively, current evidence supports continued investigation of irisin as a potential antifibrotic strategy and of its associated signaling pathways as potential therapeutic targets, while clinical translation will require resolution of these important mechanistic and pharmacological uncertainties.

## Figures and Tables

**Figure 2:**
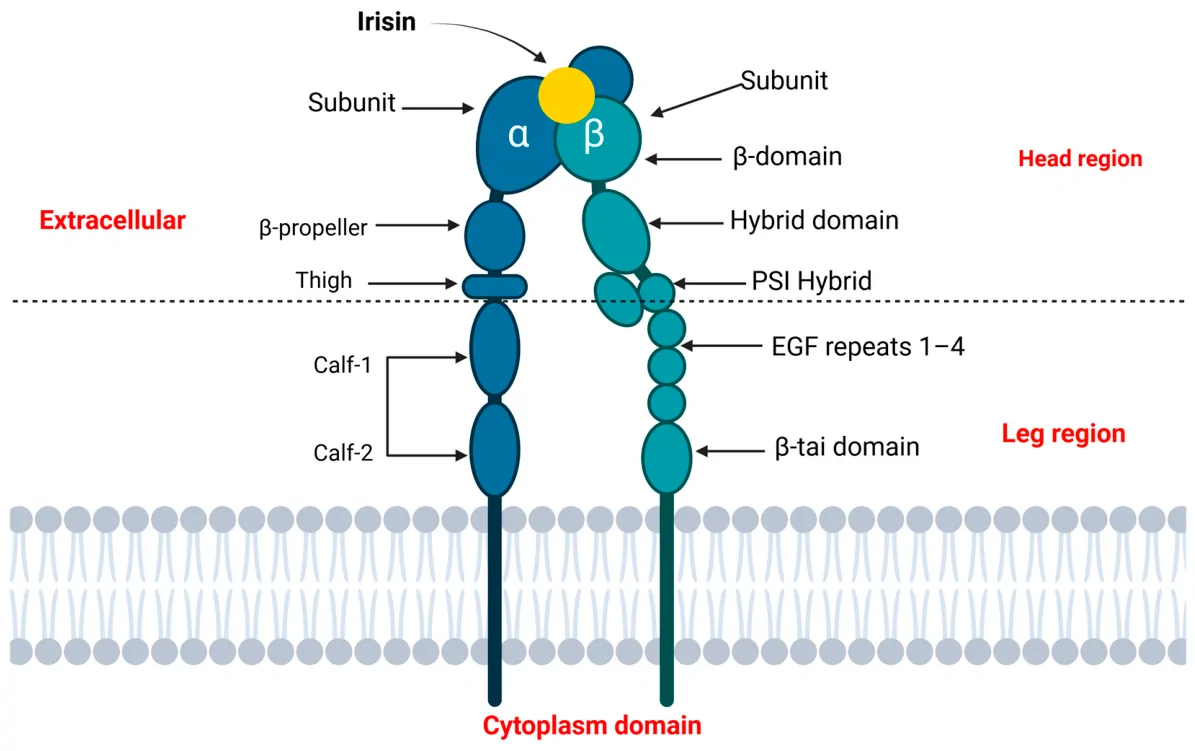
Structure of integrin αVβ5, a major functional receptor for irisin. Integrin αVβ5 is a heterodimer composed of αV and β5 subunits. Biochemical and structural studies support direct interaction between irisin and αVβ5, although additional αV-containing integrins may also participate in irisin signaling [54,55]. The extracellular region of αVβ5 comprises specialized domains involved in ligand recognition and receptor activation, whereas the transmembrane and cytoplasmic regions connect extracellular ligand binding with intracellular signaling networks [53,56,57]. Created in BioRender. Araos, P. (2026) https://BioRender.com/ethc5fn

**Figure 3:**
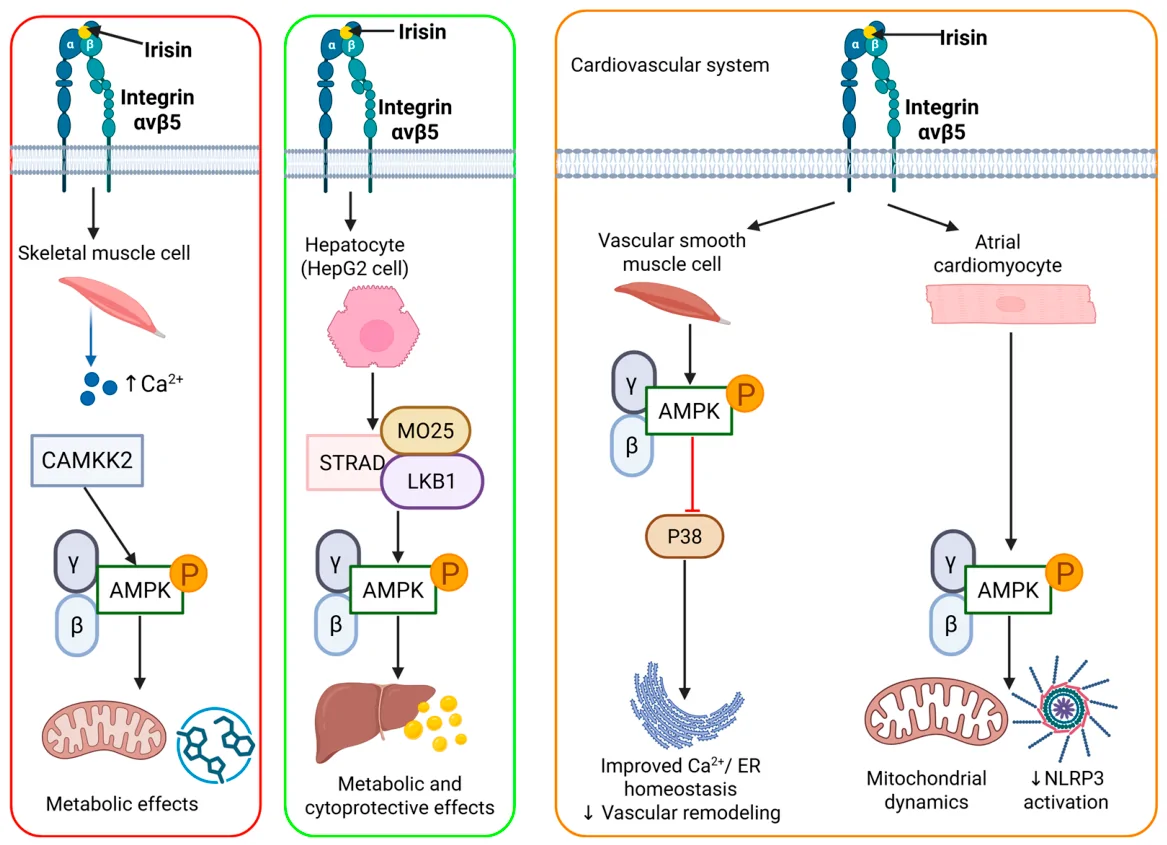
Context-dependent mechanisms of AMPK activation by irisin. Experimental evidence indicates that irisin can activate AMPK through distinct upstream mechanisms depending on the cellular context. In skeletal muscle cells, irisin increases intracellular Ca^2+^ and promotes CaMKK-dependent AMPK activation [59], whereas in human HepG2 cells, irisin-induced AMPK activation has been associated predominantly with LKB1 [60]. In the cardiovascular system, αVβ5-dependent AMPK activation has been demonstrated in vascular smooth muscle cells, where it contributes to suppression of p38 signaling and improvement of Ca^2+^ and endoplasmic reticulum homeostasis during Ang II-induced vascular remodeling [26]. FNDC5-associated AMPK activation has also been implicated in improved mitochondrial dynamics, enhanced antioxidant defenses, and reduced NLRP3 inflammasome activation in diabetes-associated atrial remodeling [29]. These findings support AMPK as an important but context-dependent mediator of irisin/FNDC5 signaling rather than as part of a uniform linear signaling cascade. ↑ indicates an increase; ↓ indicates a decrease. Created in BioRender. Araos, P. (2026) https://BioRender.com/hbsg8kf

**Figure 4:**
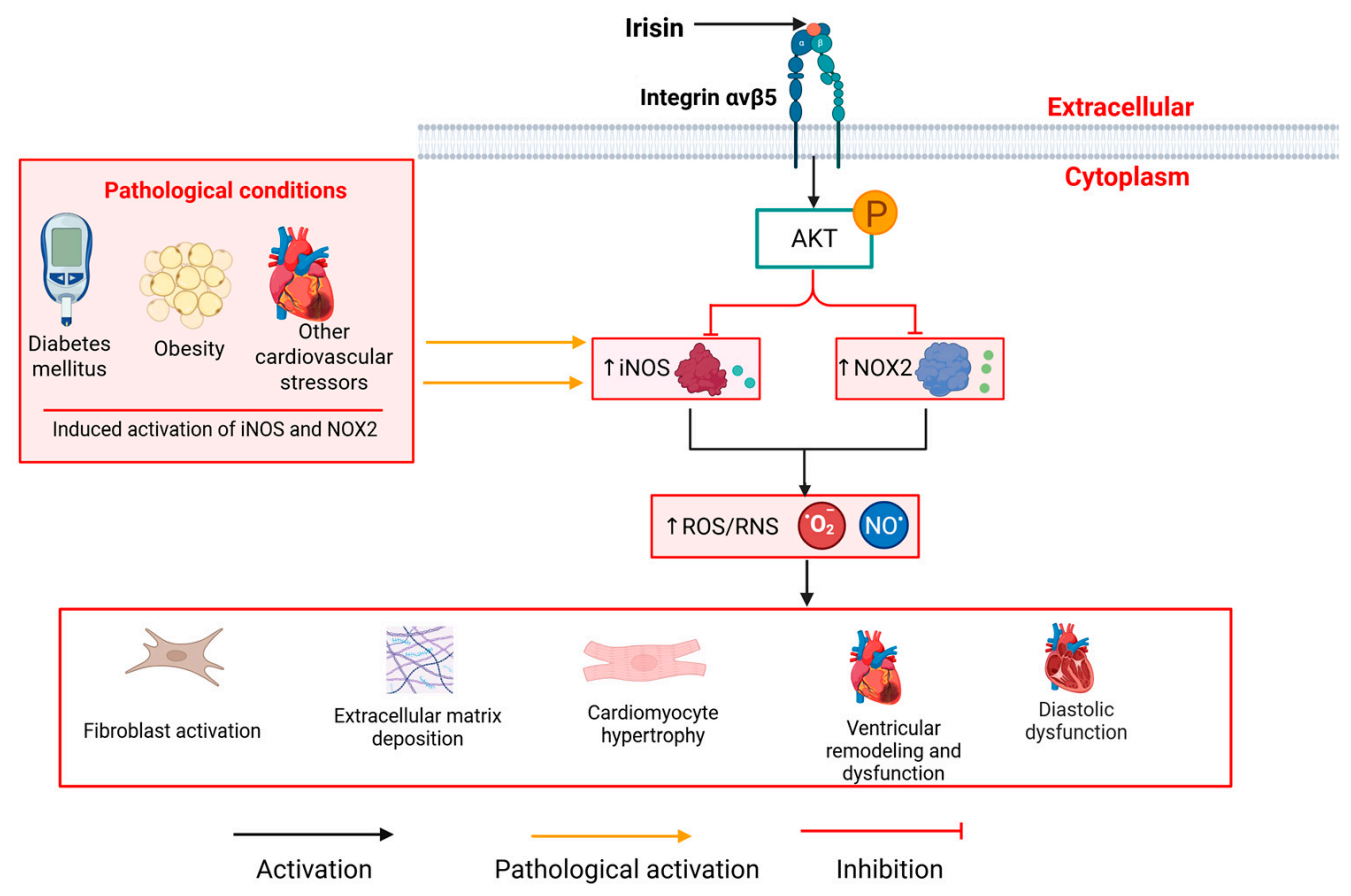
Regulation of oxidative and nitrosative stress by irisin/FNDC5 in diabetic cardiomyopathy. Irisin/FNDC5 activates integrin αVβ5-dependent AKT signaling, which is associated with reduced iNOS and NOX2 expression and attenuation of ROS and RNS accumulation. By limiting oxidative and nitrosative stress, this mechanism contributes to preservation of mitochondrial function and attenuation of pathological cardiac remodeling in experimental diabetic cardiomyopathy [24]. Created in BioRender. Araos, P. (2026) https://BioRender.com/wb3bbxb

**Figure 5:**
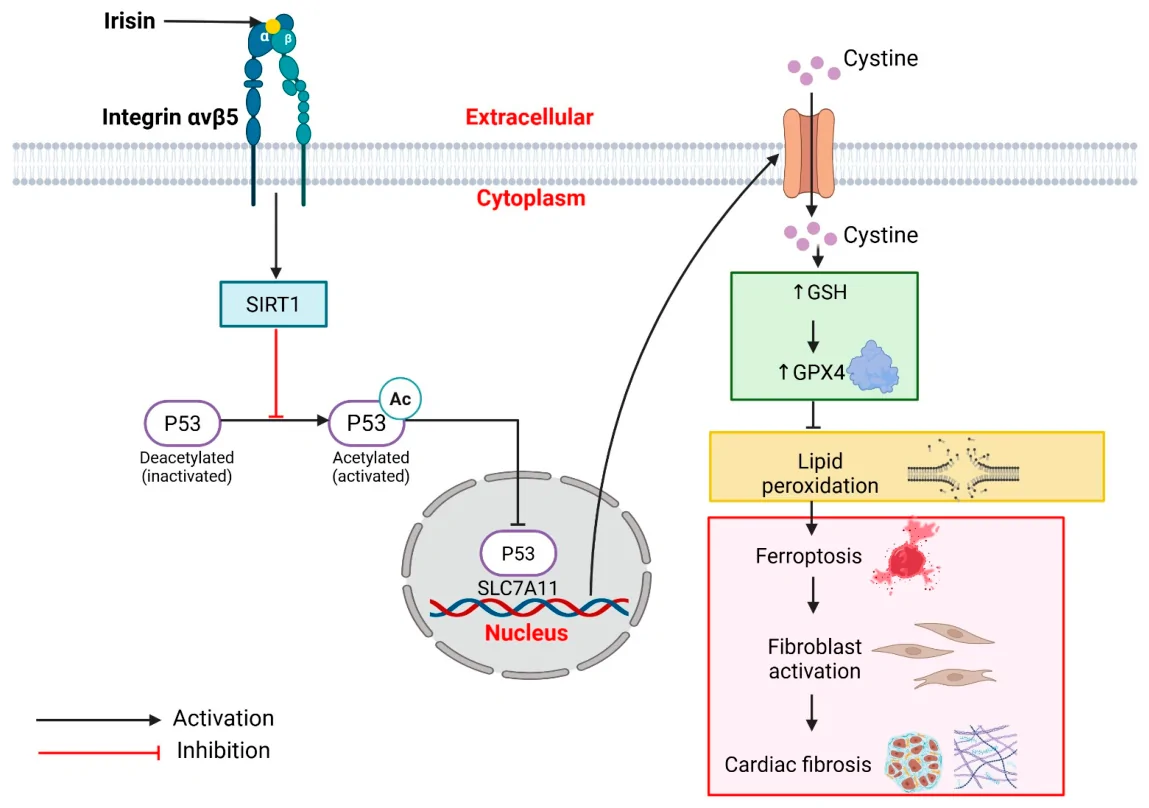
Prevention of ferroptosis through the SIRT1/p53 pathway. Irisin increases SIRT1 expression, promoting p53 deacetylation and thereby attenuating p53-dependent repression of SLC7A11. SLC7A11, a functional subunit of the cystine/glutamate antiporter system Xc^−^, facilitates cystine uptake, supporting intracellular glutathione (GSH) synthesis. GSH serves as an essential cofactor for glutathione peroxidase 4 (GPX4), which reduces membrane lipid hydroperoxides and limits lipid peroxidation. Preservation of the SLC7A11–GSH–GPX4 antioxidant system therefore reduces ferroptotic cardiomyocyte death. In experimental diabetic cardiomyopathy, irisin-mediated modulation of the SIRT1/p53 pathway attenuates ferroptosis and contributes to the reduction in pathological cardiac remodeling [30]. Created in BioRender. Araos, P. (2026) https://BioRender.com/a8abtr5.

**Figure 6:**
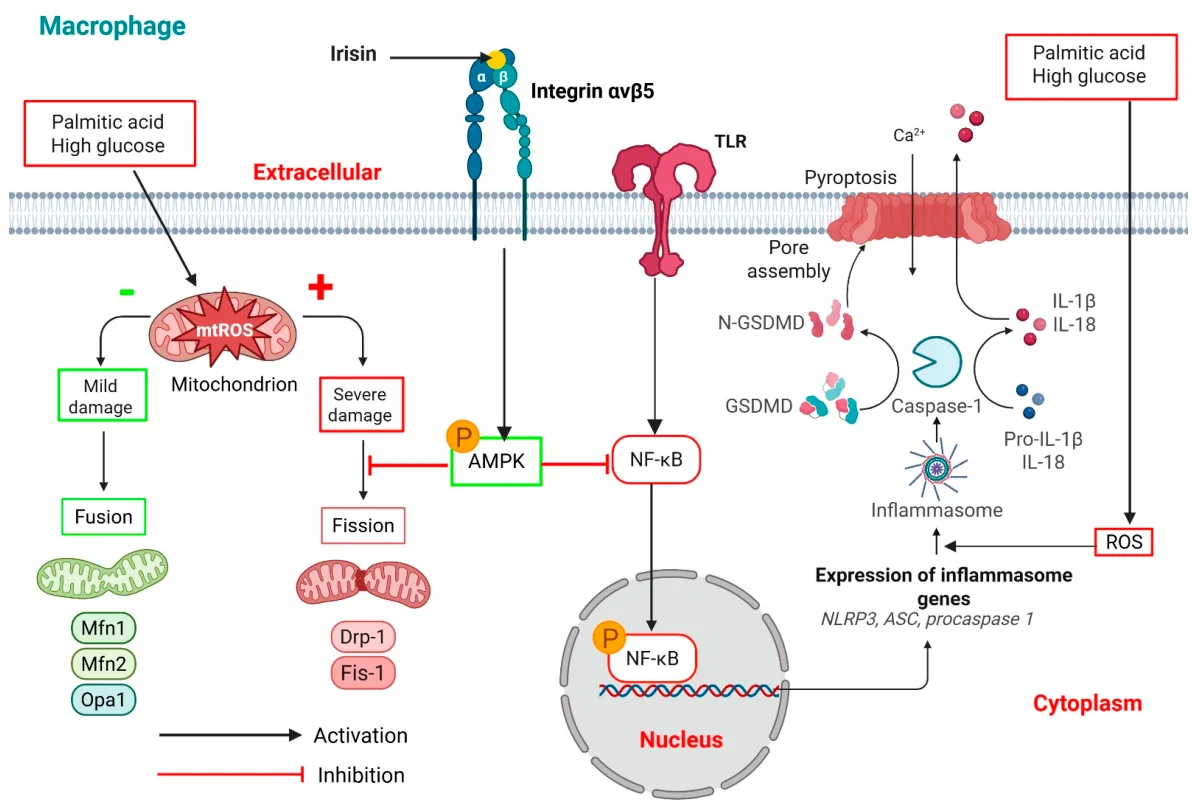
Irisin-mediated modulation of inflammatory signaling and NLRP3 inflammasome activation under metabolic stress. Metabolic stressors, including palmitic acid and high glucose, promote mitochondrial dysfunction, excessive mitochondrial reactive oxygen species (mtROS) generation, and inflammatory signaling. Irisin/FNDC5-associated AMPK activation contributes to preservation of mitochondrial homeostasis by favoring mitochondrial fusion and limiting excessive fission, thereby reducing mitochondrial oxidative stress. AMPK-associated signaling may also contribute to attenuation of NF-κB-mediated inflammatory responses. NF-κB signaling promotes the expression of inflammasome-related genes, whereas ROS provides an additional stimulus for NLRP3 inflammasome activation. Assembly of the NLRP3–ASC–caspase-1 complex promotes maturation of IL-1β and IL-18 and cleavage of gasdermin D (GSDMD), generating the pore-forming N-terminal fragment (GSDMD-N) and promoting pyroptotic cell death. Collectively, preservation of mitochondrial homeostasis and suppression of NLRP3-associated inflammatory signaling represent complementary mechanisms through which irisin/FNDC5 may attenuate inflammation and pathological cardiac remodeling [10,29,55,66]. “+” indicates a stimulatory effect; “−” indicates an inhibitory effect. Created in BioRender. Araos, P. (2026) https://BioRender.com/l5syj55.

**Figure 7:**
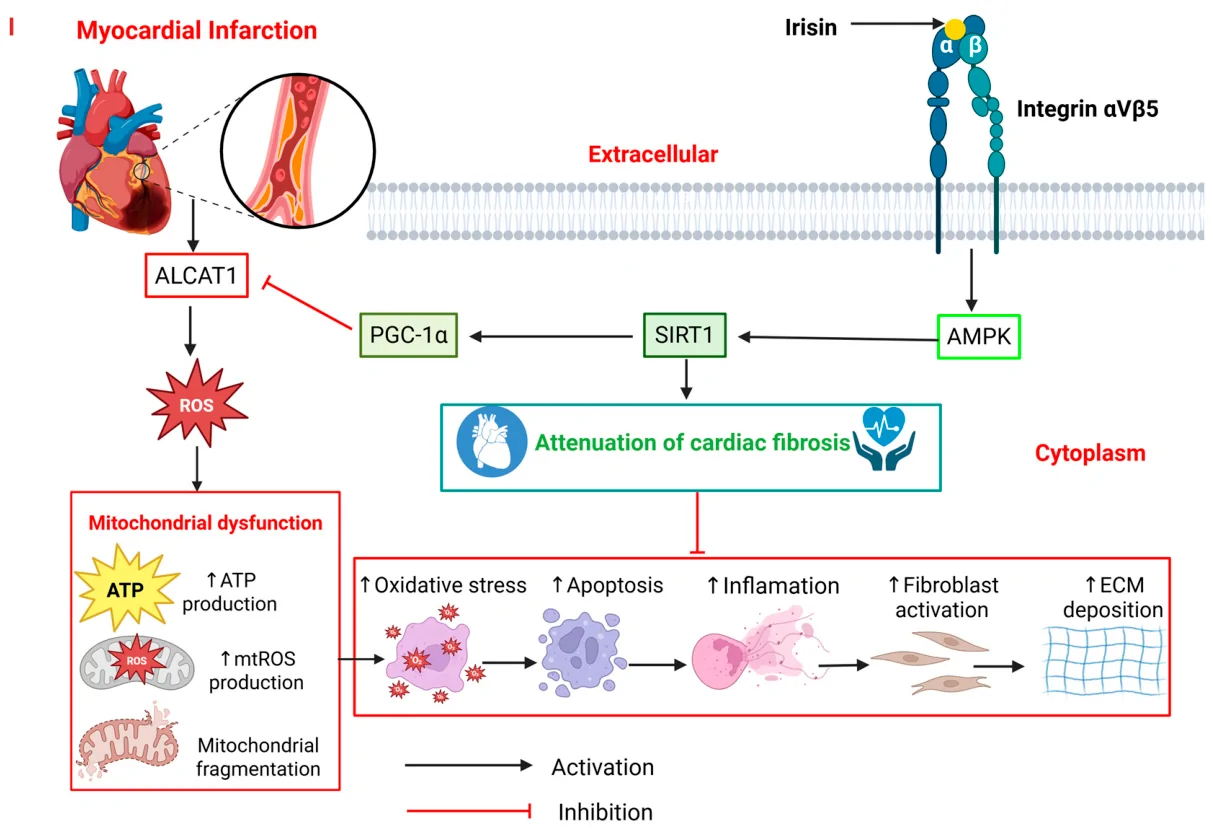
Cardioprotective mechanism of Irisin through inhibition of ALCAT1 and ROS. Following myocardial infarction, ALCAT1 (cardiolipin acyltransferase), an enzyme that aberrantly remodels mitochondrial cardiolipin and generates ROS, is induced. This mitochondrial ROS causes mitochondrial dysfunction characterized by reduced ATP production, increased mtROS, and mitochondrial fragmentation; these events, in turn, amplify oxidative stress, apoptosis, inflammation, fibroblast activation, and extracellular matrix (ECM) deposition, a sequential fibrogenic cascade. Irisin activates SIRT1 (an NAD^+^-dependent deacetylase) via AMPK, which subsequently activates PGC-1α (a master transcriptional coactivator of mitochondrial biogenesis). PGC-1α directly inhibits ALCAT1, halting the cascade at its source. The net result of this SIRT1/PGC-1α axis is the attenuation of cardiac fibrosis, thereby inhibiting the progression toward oxidative stress, apoptosis, inflammation, and fibrosis [13,45]. Created in BioRender. Araos, P. (2026) https://BioRender.com/9f0yql1.

**Figure 8:**
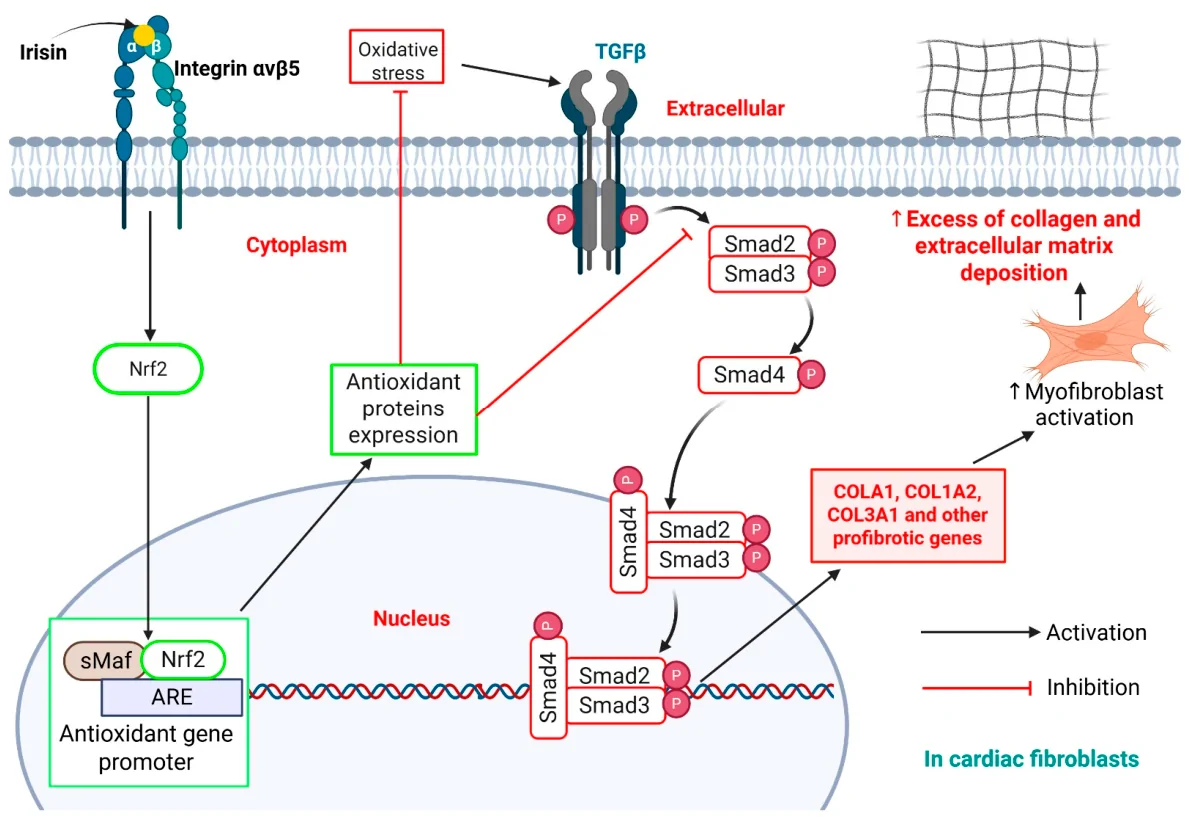
Nrf2 activation attenuates oxidative stress-dependent TGF-β1/Smad signaling and cardiac fibrogenesis. Irisin activates Nrf2-dependent antioxidant signaling. Activated Nrf2 translocates to the nucleus, where it heterodimerizes with small Maf (sMaf) proteins and binds to antioxidant response elements (AREs), promoting the expression of antioxidant and cytoprotective genes, including SOD, CAT, GPx, and HO-1. Enhanced antioxidant defenses reduce intracellular reactive oxygen species (ROS), thereby attenuating oxidative stress-dependent activation of TGF-β1/Smad2/3 signaling. In the profibrotic pathway, TGF-β1 receptor activation induces phosphorylation of Smad2/3, which associates with Smad4 and translocates to the nucleus to promote the transcription of profibrotic genes involved in myofibroblast differentiation and extracellular matrix production. By enhancing Nrf2-dependent antioxidant defenses and reducing ROS accumulation, irisin attenuates TGF-β1/Smad2/3 signaling, thereby limiting myofibroblast activation, collagen production, and extracellular matrix deposition in cardiac fibroblasts [27]. Created in BioRender. Araos, P. (2026) https://BioRender.com/qfvyxmp.

**Table 1:** Preclinical evidence supporting the cardiovascular and antifibrotic effects of irisin and experimental validation of the underlying mechanisms.

Studies	Experimental Model	Irisin-Regulated Mechanism	Mechanistic Validation/Pathway Modulation	Principal Molecular and Phenotypic Effects
**Lin et al., 2021 [24]**	T2DM cardiomyopathy: db/db mice; HG/HF-exposed H9C2 cardiomyocytes	Integrin αVβ5 → ↑ AKT; ↓ iNOS/NOX2 → ↓ oxidative/nitrosative stress	Myocardial FNDC5 overexpression and recombinant irisin administration; experimental interrogation of integrin αVβ5–AKT signaling	↑ AKT signaling; ↓ oxidative/nitrosative stress and mitochondrial apoptosis; ↓ myocardial fibrosis and hypertrophy; improved diastolic function
**Chen et al., 2019 [27]**	Ang II-induced cardiac fibrosis in mice; Ang II-stimulated cardiac fibroblasts	↑ Nrf2; ↓ ROS → ↓ TGF-β1/Smad2/3 signaling	Nrf2-targeted siRNA	↑ Nrf2 signaling; ↓ ROS, collagen synthesis, and fibroblast-to-myofibroblast differentiation; ↓ myocardial fibrosis and cardiac dysfunction
**Tang et al., 2024 [30]**	T1DM cardiomyopathy: STZ-induced diabetic mice; HG-exposed H9C2 cardiomyocytes	↑ SIRT1 → ↓ p53 acetylation → ↑ SLC7A11/GSH/GPX4 → ↓ ferroptosis	EX527 (SIRT1 inhibitor); SIRT1 siRNA; erastin (ferroptosis inducer)	↑ antioxidant/anti-ferroptotic defense; ↓ lipid peroxidation and ferroptosis; ↓ myocardial fibrosis; improved cardiac function
**Meng et al., 2024 [29]**	Diabetes-associated atrial remodeling: diabetic mice; HG+PA-exposed atrial myocytes	↑ FNDC5/AMPK signaling; ↑ SOD1/SOD2; improved mitochondrial dynamics; ↓ NLRP3 inflammasome activation	FNDC5 overexpression; assessment of AMPK signaling	↑ antioxidant capacity and mitochondrial homeostasis; ↓ NLRP3-mediated inflammation; attenuation of adverse atrial remodeling and AF susceptibility
**Li et al., 2024 [26]**	Ang II-induced hypertensive vascular remodeling: WT, FNDC5-KO, and FNDC5-overexpressing mice; Ang II-stimulated VSMCs	Integrin αVβ5 → ↑ AMPK; ↓ p38; improved Ca^2+^/ER homeostasis	αVβ5 blockade; AMPK inhibition; p38 activation; FNDC5 knockout/overexpression	↑ AMPK signaling and cellular homeostasis; ↓ ER stress, VSMC proliferation, migration, and phenotypic switching; ↓ vascular remodeling

**Abbreviations:** AF, atrial fibrillation; AKT, protein kinase B; AMPK, AMP-activated protein kinase; Ang II, angiotensin II; ER, endoplasmic reticulum; FNDC5, fibronectin type III domain-containing protein 5; GPX4, glutathione peroxidase 4; GSH, glutathione; HF, high fat; HG, high glucose; iNOS, inducible nitric oxide synthase; NLRP3, NOD-like receptor family pyrin domain-containing 3; NOX2, NADPH oxidase 2; Nrf2, nuclear factor erythroid 2-related factor 2; PA, palmitic acid; ROS, reactive oxygen species; SIRT1, sirtuin 1; STZ, streptozotocin; T1DM, type 1 diabetes mellitus; T2DM, type 2 diabetes mellitus; VSMCs, vascular smooth muscle cells; WT, wild type. ↑, increased; ↓, decreased.

**Table 2:** Comparison of established cardiovascular therapies and experimental strategies with effects on cardiac fibrotic remodeling.

Studies	Therapeutic Strategy	Principal Fibrosis-Related Mechanisms	Evidence Related to Cardiac Fibrosis	Translational Status
Brilla et al., 2000 [70]; Díez et al., 2002 [74]	ACE inhibitor/ARB	↓ Ang II/AT1-dependent profibrotic signaling; ↓ collagen synthesis/deposition	Human myocardial biopsy evidence of reduced myocardial collagen/fibrosis in hypertensive heart disease	Established cardiovascular therapy
Zannad et al., 2000 [71]; Iraqi et al., 2009 [75]	Mineralocorticoid receptor antagonists	MR blockade; modulation of collagen synthesis and ECM turnover	Reduced circulating collagen/ECM-turnover biomarkers in HF and post-MI LV dysfunction; direct histological regression of myocardial fibrosis not assessed	Established HF therapy
Zile et al., 2019 [72]	ARNI (sacubitril/valsartan)	AT1 blockade + neprilysin inhibition; modulation of profibrotic/ECM signaling	Reduced circulating biomarkers associated with ECM regulation in HFrEF; direct regression of myocardial fibrosis not established	Established HFrEF therapy
Mason et al., 2021 [73]	SGLT2 inhibitor (empagliflozin)	Metabolic, oxidative, inflammatory, and remodeling pathways	Reduced CMR-derived myocardial ECV in T2DM + CAD; direct histological antifibrotic effect not demonstrated	Established cardiovascular/HF therapy
Jiang et al., 2020 [35]; Sun et al., 2021 [18]	Xanthohumol	↑ PTEN; ↓ Akt/mTOR; ↓ TGF-β1-associated fibroblast activation	Reduced fibrosis/collagen deposition in vivo and fibroblast activation/collagen production in vitro	Preclinical
Li et al., 2019 [19]	CGRP	↑ Klotho; ↓ cardiac fibroblast senescence	Experimental attenuation of fibroblast senescence associated with cardiac fibrosis	Preclinical
Chen et al., 2019 [27]; Lin et al., 2021 [24]; Tang et al., 2024 [30]	Irisin/FNDC5	Context-dependent αVβ5/AKT, Nrf2/TGF-β1/Smad2/3, and SIRT1/p53/ferroptosis signaling	Reduced myocardial fibrosis/remodeling in Ang II-induced cardiac fibrosis and experimental diabetic cardiomyopathy; reduced fibroblast activation and collagen production	Preclinical; therapeutic efficacy in humans not established

**Abbreviations:** ACE, angiotensin-converting enzyme; ARB, angiotensin receptor blocker; ARNI, angiotensin receptor–neprilysin inhibitor; AT1, angiotensin II type 1 receptor; CGRP, calcitonin gene-related peptide; CMR, cardiac magnetic resonance; ECM, extracellular matrix; HF, heart failure; HFrEF, heart failure with reduced ejection fraction; MRA, mineralocorticoid receptor antagonist; RAAS, renin–angiotensin–aldosterone system; SGLT2, sodium–glucose cotransporter 2. ↑, increased; ↓, decreased.

## Data Availability

No new data were created or analyzed in this study. Data sharing is not applicable to this article.

## Abbreviations

ALCAT1	Acyl-CoA lysocardiolipin acyltransferase 1
AMP	Adenosine monophosphate
AMPK	AMP-activated protein kinase
ADP	Adenosine diphosphate
AT1R	Angiotensin II type 1 receptor
ATF4	Activating transcription factor 4
ATF6	Activating transcription factor 6
ATP	Adenosine triphosphate
α-SMA	Alpha-smooth muscle actin
α7 nAChR	Alpha-7 nicotinic acetylcholine receptor
Bax	Bcl-2-associated X protein
Bcl2	B-cell lymphoma 2
CaMKK2/CaMKKβ	Calcium/calmodulin-dependent protein kinase kinase 2
CDK1	Cyclin-dependent kinase 1
CDK4	Cyclin-dependent kinase 4
CHOP	C/EBP homologous protein
DAMPs	Damage-associated molecular patterns
ECM	Extracellular matrix
eIF2α	Eukaryotic initiation factor 2 alpha
FNDC5	Fibronectin type III domain-containing protein 5
GPX4	Glutathione peroxidase 4
GSH	Glutathione
HIF-1α	Hypoxia-inducible factor 1-alpha
IκB	Inhibitor of kappa B
IKK (α, β, γ)	IκB kinase complex
IL-1β, IL-4, IL-6, IL-10, IL-12, IL-17, IL-18	Interleukins
iNOS	Inducible nitric oxide synthase
IP3R	Inositol triphosphate receptor
IRE1α	Inositol-requiring enzyme 1 alpha
IRF5	Interferon regulatory factor 5
LKB1	Liver kinase B1
LOXL2	Lysyl oxidase-like 2
MMP	Matrix metalloproteinase
MO25	Mouse protein 25
mtROS	Mitochondrial reactive oxygen species
MyD88	Myeloid differentiation primary response 88
NETs	Neutrophil extracellular traps
NF-κB	Nuclear factor kappa B
NLRP3	NOD-like receptor protein 3
NOX2	NADPH oxidase 2
Nrf2	Nuclear factor erythroid 2-related factor 2
p21	Cyclin-dependent kinase inhibitor 1
p38	p38 mitogen-activated protein kinase
PERK	Protein kinase R-like endoplasmic reticulum kinase
PGC-1α	Peroxisome proliferator-activated receptor gamma coactivator 1-alpha
PI3K	Phosphoinositide 3-kinase
RAAS	Renin-angiotensin-aldosterone system
RNS	Reactive nitrogen species
ROS	Reactive oxygen species
RyR2	Ryanodine receptor 2
SARA	Smad anchor for receptor activation
SASP	Senescence-associated secretory phenotype
SERCA	Sarco/endoplasmic reticulum Ca^2+^-ATPase
SIRT1	Sirtuin 1
SLC7A11	Solute carrier family 7 member 11
SM22α	Smooth muscle protein 22-alpha
STRAD	STE20-related adaptor protein
TGF-β1	Transforming growth factor beta 1
TIMP	Tissue inhibitor of metalloproteinases
TLR4	Toll-like receptor 4
TNF-α	Tumor necrosis factor alpha
VSMCs	Vascular smooth muscle cells
